# Supplementary material for: Behavioural patterns behind the demise of the commons across different cultures
Source: R Soc Open Sci. 2020 Jul 29;7(7):201026. doi: 10.1098/rsos.201026 (PMC7428227; doi:10.1098/rsos.201026)
Supplement: Supplementary Information for: Behavioral patterns behind the demise of the commons across different cultures [file rsos201026supp1.pdf]

Supplementary Information for:

Behavioral patterns behind the demise of the commons across different cultures

M. Jusup,[1] F. Maciel-Cardoso,[2] C. Gracia-Lázaro,[3] C. Liu,[4] Z. Wang,[5] and Y. Moreno[2,6]

[1] *Tokyo Tech World Research Hub Initiative (WRHI), Institute of Innovative Research, Tokyo Institute of Technology, Tokyo 152-8552, Japan*

[2] *Institute for Biocomputation and Physics of Complex Systems (BIFI), University of Zaragoza, Zaragoza 50009, Spain*

[3] *Institute for Biocomputation and Physics of Complex Systems (BIFI), University of Zaragoza 50009, Spain*

[4] *Center for Ecology and Environmental Sciences, Northwestern Polytechnical University, Xi'an 710072, China*

[5] *Center for OPTical IMagery Analysis and Learning (OPTIMAL), Northwestern Polytechnical University, Xi'an 710072, China*

[6] *ISI Foundation, Turin 10126, Italy*

SUPPORTING METHODS

Framing and instructions

Having briefly outlined the purpose of our game experiment and the underlying context in the main text, here, we provide additional details in the form of key framing elements. This is followed by the English original of game instructions (i) from which we prepared localized Chinese and Spanish translations and (ii) through which we exposed participants to the desired framing before starting the game. The key framing elements included:

- Exact specification of the task at hand (resource exploitation), and its group nature (six people involved).
- Visualization of the resource (a virtual forest).
- Explanation that profit arises as a difference between revenue from harvested resource and harvesting cost, along with a disclosure that a depleted resource is costlier to harvest than an abundant one.
- Explanation that the resource regenerates, along with a disclosure that excessive exploitation may ultimately preclude regeneration.

To see how exactly we phrased the listed key framing elements, we refer the reader to the complete English original of game instructions displayed at the end of this section.

Computer interface

To enable participants to engage in our game experiment, we prepared a simple computer interface in o-Tree platform for laboratory, online, and field experiments [1]. The interface was accessible through any modern web browser by entering a system-generated web address into the browser's address bar. Apart from the already described instructions, the interface consisted of a gameplay page through which participants interacted with the system throughout all 50 rounds of the game (Fig. S1).

Design-wise we divided the gameplay page into three parts. Dominant was information on the resource state, which we displayed in three different forms to emphasize its importance. The most elaborate form was a cartoon visualization of the virtual forest showing trees left for cutting or leftover stumps in place of trees already cut (Fig. S1). This was supplemented with a simple vertical progress bar to the right of the cartoon and a numerical value in the status bar above the cartoon (Fig. S1). In this way, we ensured that the resource state was accessible to participants at a glance at all times.

The second largest part of the gameplay page was dedicated to performance indicators, located just below the cartoon visualization of the virtual forest (Fig. S1). We included three bar graphs that compared a player's own performance with the performance of others. Specifically, participants could see their own effort and the average effort of others in the preceding round, their own number of cut trees and the average number of trees cut by others in the preceding round, and their own average profit per round and the average per-round profit of others since the beginning of the game. All this information was accompanied with the player's own profit in the preceding round that appeared in a status bar underneath the bar graphs.

The decision-making part of the gameplay page was situated at the bottom below the performance indicators. This part contained a small input form asking participants to provide a desired number of days per week to be spent on

harvesting (Fig. S1). Inputs between 0 and 7, including decimal ones, triggered the system to automatically display the corresponding harvesting cost in a message box on the right (Fig. S1). Knowing the cost was supposed to help participants make more informed decisions because a more heavily exploited resource required more effort for the same yield. Participants had to confirm their final decision by clicking on the *Next* button just below the input form. The time allotted to decision making was 30 seconds. For orientation, a timer was always visible in a status bar on the top of the gameplay page (Fig. S1).

Clustering

We relied on the k-means technique to independently cluster data from each country. This technique is based on minimizing the within-cluster sum of squares or, equivalently, the pairwise squared distances between the same-cluster points. The minimization algorithm requires defining a distance metric in a d-dimensional space, where d is the number of characteristics used for clustering. Here, we opted for the Euclidean distance and $d = 4$ dimensions: cumulative effort in the first half, cumulative effort in the second half, total profit in the first half, and total profit in the second half of the game. There was no need to standardize data points prior to clustering because the selected characteristics are roughly of the same scale.

Although the k-means technique is rather well-known, we present its mathematical formulation for completeness. Let $X = \{\mathbf{X}_1, \dots, \mathbf{X}_n\}$ be a set of n observations, where each observation $\mathbf{X}_p$ is a d-dimensional vector. The goal of the k-means technique is to partition these observations into $k \leq n$ sets $S_1, \dots, S_k$, $S_q = \{\mathbf{X}_p \in X : p \in I_q \subseteq \{1, \dots, n\}\}$, in a manner that minimizes the within-cluster sum of squares

$$\underset{S_1,\dots,S_k}{\operatorname{argmin}} \sum_{q=1}^{k} \sum_{\mathbf{X}\in S_q} \|\mathbf{X} - \boldsymbol{\xi}_q\|^2.$$

Here, $\boldsymbol{\xi}_q$ is the centroid of S_q. Intuitively we thus have that within-cluster points should be as close as possible to their centroid. Each observation is, in fact, assigned to a cluster whose centroid is the closest, implying that the resulting partitioning of the data space represents a Voronoi diagram. An alternative, but mathematically equivalent approach to k-means clustering is to minimize the pairwise squared distances between same-cluster points

$$\underset{S_1,\dots,S_k}{\operatorname{argmin}} \sum_{q=1}^{k} \frac{1}{2\,|S_q|} \sum_{\mathbf{X},\,\mathbf{Y}\in S_q} \|\mathbf{X} - \mathbf{Y}\|^2.$$

Solving the k-means clustering problem for optimal solution is generally an NP-hard problem. However, a number of heuristic algorithms are available. We used the Hartigan-Wong algorithm [2] as implemented in the `kmeans` function of the `stats` package in R statistical software.

Behavioral regression model

For mechanistic insights into decision making in the context of common-pool resource exploitation, we constructed a statistical regression model of participant behavior. This model comprised three types of explanatory variables:

- The virtual forest's state was included because we expected participants to exhibit different behaviors when the resource is abundant as opposed to when the resource is depleted.

- Lagged own efforts were included to account for potential autocorrelations in the play of individual participants; positive autocorrelations, in particular, would be an indication of decision-making "inertia" whereby high (resp., low) past efforts increase the likelihood of high (resp., low) present effort.
- Lagged average efforts of others were included to account for potential cross-correlations as a reflection of mutual influences between participants.

Model parameters, i.e., regression coefficients, accompanying these three types of explanatory variables were kept constant among participants from a given country, thus characterizing a collective behavioral mode. More technically, the total variance in the data comprises contributions from individual variances and mutual covariances. A model with a constant set of parameter values for all individuals is more likely to capture the latter contribution, while the former is captured to the extent that individuals mimic the collective.

Alongside constant model parameters for all participants from a given country, we allowed individual differences to enter the model via participant-specific constant terms, called fixed effects, and via participant-specific residual variances. We interpreted the former as individualistic propensities to exert effort irrespective of the state of the explanatory variables. Accordingly, players with larger fixed effects were more likely to cut trees even if the number of trees left for logging was small, or even if other players refrained from logging. Residual variances, by contrast, quantified individualistic propensities to randomly vary effort. We introduced participant-specific residual variances because we expected that human participants would exhibit a wide spectrum of behaviors. We anticipated that some players would stick with previous own decisions, while others would explore all options more freely. With these ideas in mind, a general model formulation was

$$T_i\left(t\right) = \beta_R R\left(t\right) + \sum_{s=1}^{S_1} \beta_T^{-s} T_i\left(t-s\right) + \sum_{s=1}^{S_2} \beta_{\langle T\rangle}^{-s} \left\langle T\left(t-s\right)\right\rangle + \beta_i + \epsilon_i\left(t\right), \tag{S1}$$

where dependent variable $T_i\left(t\right)$ is the ith player's effort in round t. Among the three types of explanatory variables, $R\left(t\right)$ is the virtual forest's state in round t, $T_i\left(t-s\right)$ is the ith player's lagged effort s rounds prior to t, and $\left\langle T\left(t-s\right)\right\rangle$ is the lagged average effort of others, also s rounds prior to t. The numbers of lagged terms in the model, S_1 and S_2, were unknown prior to parameter estimation. Quantity β_i is the model's constant term, i.e., a fixed effect specific to the ith player. Finally, $\epsilon_i\left(t\right)$ are the model's normally distributed residuals with zero mean and residual variance σ_i^2, again specific to the ith player. Assuming the normal distribution here implied a lack of autocorrelative structure in residuals. This was reasonable given that the lagged own efforts in Eq. (S1) should account for potential autocorrelations in player decisions.

Parameter estimation

To estimate the model parameters appearing in Eq. (S1), i.e., β_R, β_T^{-s}, $\beta_{\langle T\rangle}^{-s}$, and β_i, as well as residual variances σ_i^2, we resorted to an appropriate variant of the maximum likelihood method. Specifically, the log-likelihood function to maximize was

$$\log L_{NT}\left(\boldsymbol{\beta}, \boldsymbol{\sigma^2}\right) = \sum_{i=1}^{N}\sum_{t=1}^{T} \log l_{NT}\left(\boldsymbol{\beta}, \boldsymbol{\sigma^2}\right) = -\frac{1}{2}\sum_{i=1}^{N}\sum_{t=1}^{T}\left[\log\left(2\pi\sigma_i^2\right) + \frac{\epsilon_i^2\left(t\right)}{\sigma_i^2}\right], \tag{S2}$$

where $\boldsymbol{\beta} = \left(\beta_R, \beta_T^{-s}, \beta_{\langle T\rangle}^{-s}, \beta_1, \ldots, \beta_N\right)$ and $\boldsymbol{\sigma^2} = \left(\sigma_1^2, \ldots, \sigma_N^2\right)$ are symbolic notation for all model parameters and participant-specific residual variances, respectively, N is the number of players in a given country, and T is the

number of rounds played. This type of log-likelihood function arises naturally when working with panel data, i.e., observations of multiple individuals over a period of time, as was the case herein. We assumed that the residual variance was constant for each player, but that players differed from one another, thus giving rise to a continuous spectrum of possible values. An additional assumption implied by log-likelihood function (S2) was that residual covariances have the form $\mathrm{E}\epsilon_i\epsilon_j = \delta_{ij}\sigma_i\sigma_j$, where δ_{ij} is the Kronecker delta, i.e., $\delta_{ij} = 1$ if $i = j$ and $\delta_{ij} = 0$ otherwise. Similarly as with autocorrelations, this assumption was reasonable given that the lagged average efforts of others in Eq. (S1) should account for potential cross-correlations in player decisions.

To estimate parameter values $\hat{\boldsymbol{\beta}}$ that maximize log-likelihood function (S2), we used a generalized least squares estimator described by Hayashi [3], but only after casting the regression problem into a suitable form (see *Regression analysis* below). This estimator is implicit in the sense that residual variances to be estimated, $\hat{\boldsymbol{\sigma}}^{\mathbf{2}}$, appear on the right side of the estimation equation. We therefore implemented an iterative numerical algorithm proposed by Amemiya [4]. Furthermore, to calculate robust confidence intervals, we approximated the covariance matrix of pair $\left(\hat{\boldsymbol{\beta}}, \hat{\boldsymbol{\sigma}}^{\mathbf{2}}\right)$ with the sandwich estimator [5]. For the purpose of regression diagnostics, we confirmed the validity of these confidence intervals with a bootstrap procedure, as well as tested parameter estimability under model misspecification (Supplementary Note 4).

Among goodness-of-fit measures, the coefficient of determination R^2 is ubiquitous and offers intuitive appeal. We relied on a generalized definition of R^2 due to Nagelkerke [6]

$$R^2 = 1 - \exp\left[-\frac{2}{NT}\left(\log L_{NT}\left(\hat{\boldsymbol{\beta}}, \hat{\boldsymbol{\sigma}}^{\mathbf{2}}\right) - \log L_{NT}\left(\hat{\boldsymbol{\beta}}_{\mathbf{0}}, \hat{\boldsymbol{\sigma}}^{\mathbf{2}}_{\mathbf{0}}\right)\right)\right], \tag{S3}$$

where $\log L_{NT}\left(\hat{\boldsymbol{\beta}}_{\mathbf{0}}, \hat{\boldsymbol{\sigma}}^{\mathbf{2}}_{\mathbf{0}}\right)$ is the log-likelihood of the null model for which $\hat{\boldsymbol{\beta}}_{\mathbf{0}} = (0, 0, 0, \hat{\beta}_0, \ldots, \hat{\beta}_0)$, while $\hat{\boldsymbol{\sigma}}^{\mathbf{2}}_{\mathbf{0}}$ are the corresponding participant-specific residual variances. The said intuitive appeal of this definition stems from the fact that (i) $R^2 > 0$ for any model with free parameters that fits the data better than the null model, (ii) $R^2 = 1$ only if the fit is perfect, and (iii) R^2 is the proportion of explained variance in the data [6]. Furthermore, Eq. (S3) explicitly incorporates the ratio of likelihood functions, thus showing that R^2 is closely related to the likelihood ratio test for the significance of regression [7], which we exploited to demonstrate that our results are indeed significant (Supplementary Note 4).

Regression analysis

Here, we detail the elements of the regression analysis needed to estimate the model's parameters. Specifically, we describe how to (i) cast the regression problem into the most convenient form, (ii) numerically calculate the parameter values, and (iii) find robust confidence intervals using the sandwich estimator.

Let $\{i \in \mathbb{N} : 1 \leq i \leq N\}$ be an index set for players and $\{t \in \mathbb{N} : 1 \leq t \leq T\}$ an index set for time. We collected dependent variables y_{it} $(= T_{it})$, each of which represents the ith player's effort at moment t, in a T-vector of observations $\mathbf{y}_i = (y_{i1}, \ldots, y_{iT})^{\mathrm{tr}}$, where "tr" denotes the transpose operation, i.e., the ith player is represented by column-vector $\mathbf{y}_i$ containing T observations. Similarly, we collected the predictor variables at moment t in vector $\mathbf{X}_{it} = (R_{it}, T_{i,t-1}, T_{i,t-2}, \ldots, \langle T_{i,t-1}\rangle, 1)^{\mathrm{tr}}$, where R_{it} is the virtual forest's state, $T_{i,t-1}$, $T_{i,t-2}$, etc. are the past efforts, $\langle T_{i,t-1}\rangle$ is the past average effort of players excluding the ith player, and 1 is included to capture the fixed effects. We subsequently created a matrix of observations for the ith player with T rows $\mathbf{X}_i = (\mathbf{X}_{i1}, \ldots, \mathbf{X}_{iT})^{\mathrm{tr}}$. Finally, we

set the vector of parameters to $\boldsymbol{\beta}_i = (\beta_{i1}, \beta_{i2}, \ldots, \beta_{ip}, \beta_i)^{\text{tr}}$. With these definitions, the regression equation for the ith player became $\mathbf{y}_i = \mathbf{X}_i\boldsymbol{\beta}_i + \boldsymbol{\epsilon}_i$. We assumed that residual vectors $\boldsymbol{\epsilon}_i = (\boldsymbol{\epsilon}_{i1}, \ldots, \boldsymbol{\epsilon}_{iT})^{\text{tr}}$ are normally distributed with covariance matrices $V\boldsymbol{\epsilon}_i = E\boldsymbol{\epsilon}_i\boldsymbol{\epsilon}_i^{\text{tr}} = \sigma_i^2\mathbf{I}$. Here, $\mathbf{I}$ is the $T \times T$ identity matrix. We had no basis on which to assume constancy of the variance across different players; it generally holds that $\sigma_i \neq \sigma_j$. Defining $\mathbf{y} = (\mathbf{y}_1^{\text{tr}}, \ldots, \mathbf{y}_N^{\text{tr}})^{\text{tr}}$, $\boldsymbol{\beta} = (\boldsymbol{\beta}_1^{\text{tr}}, \ldots, \boldsymbol{\beta}_N^{\text{tr}})^{\text{tr}}$, $\boldsymbol{\epsilon} = (\boldsymbol{\epsilon}_1^{\text{tr}}, \ldots, \boldsymbol{\epsilon}_N^{\text{tr}})^{\text{tr}}$, and $\mathbf{X} = \mathbf{X}_1 \oplus \ldots \oplus \mathbf{X}_N$, the regression equations for all players turned into $\mathbf{y} = \mathbf{X}\boldsymbol{\beta} + \boldsymbol{\epsilon}$. Here, $\mathbf{y}$ and $\boldsymbol{\epsilon}$ are column vectors with TN entries, $\boldsymbol{\beta}$ is a column vector with $N(p+1)$ entries, and matrix $\mathbf{X}$ is a direct sum of $\mathbf{X}_1, \ldots, \mathbf{X}_N$, i.e., $\mathbf{X}$ is a diagonal block matrix with the first block being $\mathbf{X}_1$, the second block being $\mathbf{X}_2$, etc. The covariance matrix of $\boldsymbol{\epsilon}$ is given by the Kronecker product, $V\boldsymbol{\epsilon} = E\boldsymbol{\epsilon}\boldsymbol{\epsilon}^{\text{tr}} = \boldsymbol{\sigma^2} \otimes \mathbf{I}$, where $\boldsymbol{\sigma^2} = \operatorname{diag}\left(\sigma_1^2, \ldots, \sigma_N^2\right)$, i.e., $\boldsymbol{\epsilon}$ is a diagonal matrix in which entries σ_1^2, σ_2^2, etc. repeat T times each. In general, the regression model we have just described belongs to a class of seemingly unrelated regression (SUR) models [4].

We appended the SUR model with constraints

$$
\begin{aligned}
&\beta_{11} = \ldots = \beta_{i1} = \ldots = \beta_{N1} = \beta_R, \\
&\beta_{12} = \ldots = \beta_{i2} = \ldots = \beta_{N2} = \beta_T^{-1}, \\
&\beta_{13} = \ldots = \beta_{i3} = \ldots = \beta_{N3} = \beta_T^{-2}, \\
&\quad\vdots \\
&\beta_{1p} = \ldots = \beta_{ip} = \ldots = \beta_{Np} = \beta_{\langle T\rangle}^{-1},
\end{aligned}
$$

where β_R, β_T^{-1}, β_T^{-2}, etc. and $\beta_{\langle T\rangle}^{-1}$ are constant parameters as defined in Eq. (S1). Although somewhat counter-intuitive at first, we cast these constraints in matrix form $\mathbf{Q}^{\text{tr}}\boldsymbol{\beta} = \mathbf{0}$ to make the implementation easier. Matrix $\mathbf{Q}$ is an $N(p+1) \times p(N-1)$ matrix. One way to set up this matrix was for the first column to indicate that the first parameter for player 1 equals the first parameter for player 2 ($\beta_{11} = \beta_{21}$), the second column to indicate that the first parameter for player 2 equals the first parameter for player 3 ($\beta_{21} = \beta_{31}$), and so on until column $N-1$ ($\beta_{N-1,1} = \beta_{N1}$). Columns N to $2(N-1)$ did the same for the second parameter (β_{*2}), and so on until columns $(p-1)N$ to $p(N-1)$ for the pth parameter (β_{*p}). To finally implement the described constraints we had to define another $N(p+1) \times (p+N)$ matrix $\mathbf{R}$ such that the block-matrix $\mathbf{A} = [\mathbf{Q}\mathbf{R}]^{\text{tr}}$ is non-singular and $\mathbf{R}^{\text{tr}}\mathbf{Q} = \mathbf{0}$. Putting $\boldsymbol{\gamma} = \mathbf{A}\boldsymbol{\beta}$, the SUR regression model turned into $\mathbf{y} = \mathbf{X}\mathbf{A}^{-1}\boldsymbol{\gamma} + \boldsymbol{\epsilon}$. In this equation, the parameter vector $\boldsymbol{\gamma}$ by definition consists of two parts, $\boldsymbol{\gamma}_1 = \mathbf{Q}^{\text{tr}}\boldsymbol{\beta} = \mathbf{0}$ and $\boldsymbol{\gamma}_2 = \mathbf{R}^{\text{tr}}\boldsymbol{\beta}$. Only the latter part, which is an $(p+N)$-vector, remained unspecified. The number of entries in $\boldsymbol{\gamma}_2$ reflected the fact that our model had p parameters and N fixed effects, i.e., one fixed effect per player. Using property $\mathbf{A}^{-1} = \left[\mathbf{Q}\left(\mathbf{Q}^{\text{tr}}\mathbf{Q}\right)^{-1}\mathbf{R}\left(\mathbf{R}^{\text{tr}}\mathbf{R}\right)^{-1}\right]$, we made additional transformation $\mathbf{X}' = \mathbf{X}\mathbf{R}\left(\mathbf{R}^{\text{tr}}\mathbf{R}\right)^{-1}$ to ultimately reformulate the initial regression problem as $\mathbf{y} = \mathbf{X}'\boldsymbol{\gamma}_2 + \boldsymbol{\epsilon}$. The last equation was no longer a SUR model, because matrix $\mathbf{X}'$ could not be expressed as a direct sum of other matrices. Revisiting the definitions of models based on covariance matrices revealed that we were facing a heteroscedastic model with a constant variance within subsets of the sample [4].

A log-likelihood function corresponding to the described regression problem is specified in Eq. (S2). To maximize this log-likelihood function, we first defined a generalized least squares estimator [3] in the form

$$
\hat{\boldsymbol{\gamma}}_2^{\text{GLS}} = \left[\mathbf{X}'^{\,\text{tr}}\left(V\boldsymbol{\epsilon}\right)^{-1}\mathbf{X}'\right]^{-1}\mathbf{X}'^{\,\text{tr}}\left(V\boldsymbol{\epsilon}\right)^{-1}\mathbf{y}. \tag{S4}
$$

This expression, unlike the expression for the more common ordinary least squares estimator, is not an explicit equation whose evaluation immediately results in parameter estimates. Instead, covariance matrix $V\boldsymbol{\epsilon}$ is unknown and needs to be estimated from data alongside parameter vector γ_2. To this end, we employed an iterative numerical algorithm as follows [4]. The algorithm is initialized with the parameter estimates obtained from the ordinary least squares estimators, $\hat{\boldsymbol{\gamma}}_2^{\rm OLS} = \left[\mathbf{X}'^{\rm tr}\mathbf{X}'\right]^{-1}\mathbf{X}'^{\rm tr}\mathbf{y}$ and $\hat{\sigma}_i^2 = \hat{\boldsymbol{\epsilon}}_i^{\rm tr}\hat{\boldsymbol{\epsilon}}_i$. Here, the residual vector estimator is $\hat{\boldsymbol{\epsilon}}_i = \mathbf{y}_i - \mathbf{X}_i'\hat{\boldsymbol{\gamma}}_{2,i}^{\rm OLS}$. Vector $\mathbf{y}_i$ (resp., $\hat{\boldsymbol{\gamma}}_{2,i}^{\rm OLS}$) contains the elements of $\mathbf{y}$ (resp., $\hat{\boldsymbol{\gamma}}_2^{\rm OLS}$) that correspond to the ith player, while similarly matrix $\mathbf{X}_i'$ contains the rows of $\mathbf{X}'$ that correspond to the ith player. Estimates $\hat{\sigma}_i^2$ are then used to construct the covariance matrix according to $V\boldsymbol{\epsilon} = \boldsymbol{\sigma^2} \otimes \mathbf{I}$, which inserted into Eq. (S4) yields the first iteration value of $\hat{\boldsymbol{\gamma}}_2^{\rm GLS}$. Subsequent iterations differ from the first one only in that $\hat{\boldsymbol{\epsilon}}_i = \mathbf{y}_i - \mathbf{X}_i'\hat{\boldsymbol{\gamma}}_{2,i}^{\rm GLS}$, where $\hat{\boldsymbol{\gamma}}_{2,i}^{\rm GLS}$ follows from a preceding iteration. The algorithm stops when all the elements of $\hat{\boldsymbol{\gamma}}_2^{\rm GLS}$ change less than desired precision δ between two consecutive iterations. We used $\delta = 10^{-12}$ throughout.

Once we had estimated the parameter values, the first among the remaining tasks was to estimate the corresponding 95% confidence intervals. We relied on a theorem of the maximum likelihood theory stating that estimator $\hat{\boldsymbol{\gamma}}_2$ asymptotically has a normal distribution with mean $\boldsymbol{\gamma}_2$ and covariance matrix $V\hat{\boldsymbol{\gamma}}_2$, the elements of which can be approximated by the sandwich estimator, $\mathbf{S}_{NT}$, defined as follows [5, 8]. Let $\boldsymbol{\theta} = \left(\boldsymbol{\gamma}_2^{\rm tr}, (\boldsymbol{\sigma^2})^{\rm tr}\right)^{\rm tr}$, then

$$\mathbf{S}_{NT}\left(\hat{\boldsymbol{\theta}}\right) = \mathbf{A}_{NT}^{-1}\left(\hat{\boldsymbol{\theta}}\right)\mathbf{B}_{NT}\left(\hat{\boldsymbol{\theta}}\right)\mathbf{A}_{NT}^{-1}\left(\hat{\boldsymbol{\theta}}\right),$$

where matrices $\mathbf{A}_{NT}\left(\hat{\boldsymbol{\theta}}\right)$ and $\mathbf{B}_{NT}\left(\hat{\boldsymbol{\theta}}\right)$ are symbolically given by

$$\mathbf{A}_{NT}\left(\hat{\boldsymbol{\theta}}\right) = \left[\sum_{i=1}^{N}\sum_{t=1}^{T}\left.\frac{\partial^2 \log l_{NT}}{\partial\boldsymbol{\theta}\partial\boldsymbol{\theta}^{\rm tr}}\right|_{\hat{\boldsymbol{\theta}}}\right],$$

$$\mathbf{B}_{NT}\left(\hat{\boldsymbol{\theta}}\right) = \left[\sum_{i=1}^{N}\sum_{t=1}^{T}\left.\frac{\partial \log l_{NT}}{\partial\boldsymbol{\theta}}\right|_{\hat{\boldsymbol{\theta}}}\left.\frac{\partial \log l_{NT}}{\partial\boldsymbol{\theta}^{\rm tr}}\right|_{\hat{\boldsymbol{\theta}}}\right],$$

with l_{NT} being the probability density function for model residuals appearing in Eq. (S2). Remembering that our main concern was parameter vector $\boldsymbol{\beta}$, its estimator $\hat{\boldsymbol{\beta}}$, and the corresponding covariance matrix $V\hat{\boldsymbol{\beta}}$, we proceeded to find the link between $V\hat{\boldsymbol{\beta}}$ and $V\hat{\boldsymbol{\gamma}}_2$. From the definition of $\boldsymbol{\gamma}_2$, it followed that $\hat{\boldsymbol{\beta}} = \mathbf{R}\left(\mathbf{R}^{\rm tr}\mathbf{R}\right)^{-1}\hat{\boldsymbol{\gamma}}_2$. This equality shows that estimator $\hat{\boldsymbol{\beta}}$ is also asymptotically normal with covariance matrix $V\hat{\boldsymbol{\beta}} = \mathbf{R}\left(\mathbf{R}^{\rm tr}\mathbf{R}\right)^{-1}V\hat{\boldsymbol{\gamma}}_2\left(\mathbf{R}^{\rm tr}\mathbf{R}\right)^{-1}\mathbf{R}^{\rm tr}$. The diagonal elements of $V\hat{\boldsymbol{\beta}}$ contained information on the standard errors of the estimated parameters. We used this information to calculate the corresponding z-scores and thereafter the 95% parameter confidence intervals.

Instructions

Thank you for participating in this experiment, which is a part of a research project wherein we try to understand how individuals make decisions. Relax and do not worry about meeting any expectations. The experiment will begin shortly. Please keep quiet until the end, turn off your cell phone, and remember that the use of any material foreign to the experiment is not allowed (including pen, pencil, or paper). If you need help, raise your hand and wait to be assisted. Please do not ask any questions aloud.

You participate along with five other people with whom you will interact according to the rules explained below. The rules are the same for all players. You will never know whom you are interacting with, and nobody will know if you are in their experiment. People around you in the room are not necessarily those who are participating in your experiment. The experiment will last around 40 minutes.

The experiment consists of an undetermined number of rounds. Your earnings will depend on your own decisions and those of the other participants. Your total earnings in this experiment will comprise cumulative earnings from all rounds plus a show-up fee of 5 €. You will receive your earnings when the experiment finishes. For convenience, the total earnings are rounded to the nearest 50 cents.

You will access the experimental interface after reading these instructions. All participants must finish reading before starting.

The game: 1st round

This is the screen you will see in the first round (this screenshot is only an example):

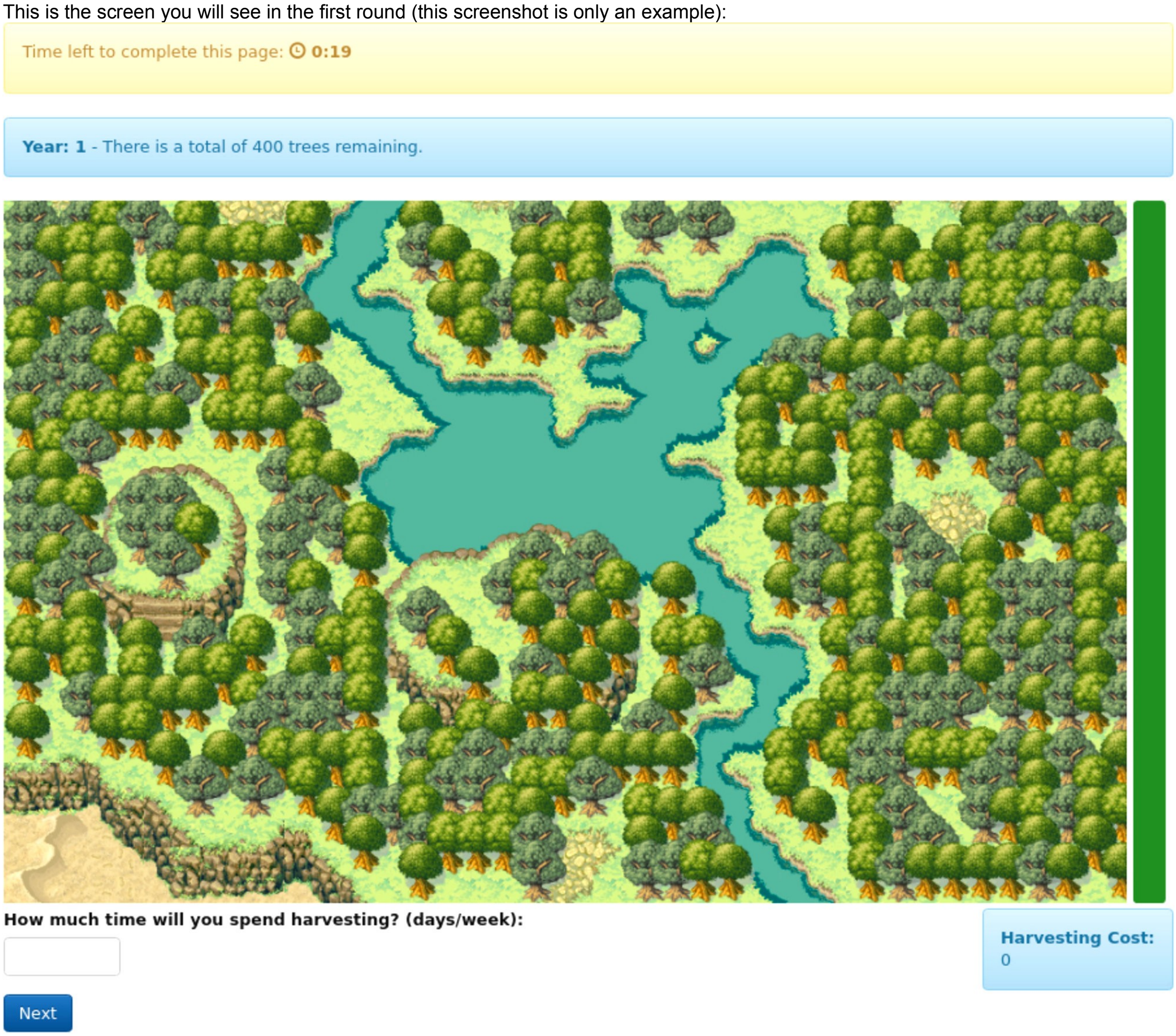

A group of 6 people (you and 5 co-players) are allowed to exploit a resource, i.e., trees shown in the screenshot. The green bar on the right side represents the fraction of remaining trees. The number of trees in the forest is shown on top. You and the rest of the players simultaneously have to make a decision, in particular you are required to input the amount of time (days/week) you are willing to spend exploiting the resource, which must be between 0 and 7 days/week. Decimal numbers are allowed (e.g., 5.2). You will have 30 seconds to post your choice. After these 30 seconds, if you didn't choose, the system will choose for you, but you will be able to make your subsequent choices normally. Do not worry, 30 seconds are more than enough to make a choice. Stay focused and do not waste time.

The effort invested in harvesting has a cost. You can see the cost associated with your choice at the bottom right of the screen. The profit you get in one round is the result of subtracting this harvesting cost from the revenue derived from harvested trees. Note that, as the number of trees gets lower, you need more effort to harvest one tree. Conversely, more trees make harvesting easier. In addition, harvested trees are replaced with new ones, but these new trees need some time to grow before becoming available for harvesting. Beware that with a high exploitation the forest may reach a critical state beyond which trees will not be able to regenerate.

Once all the players have posted their decisions, a program simulates exploitation, as well as the natural regrowth of trees during one year. The results will be displayed on screen in the next round. Each round represents a year.

The game: subsequent rounds

This is the screen you will see in subsequent rounds (this screenshot is only an example):

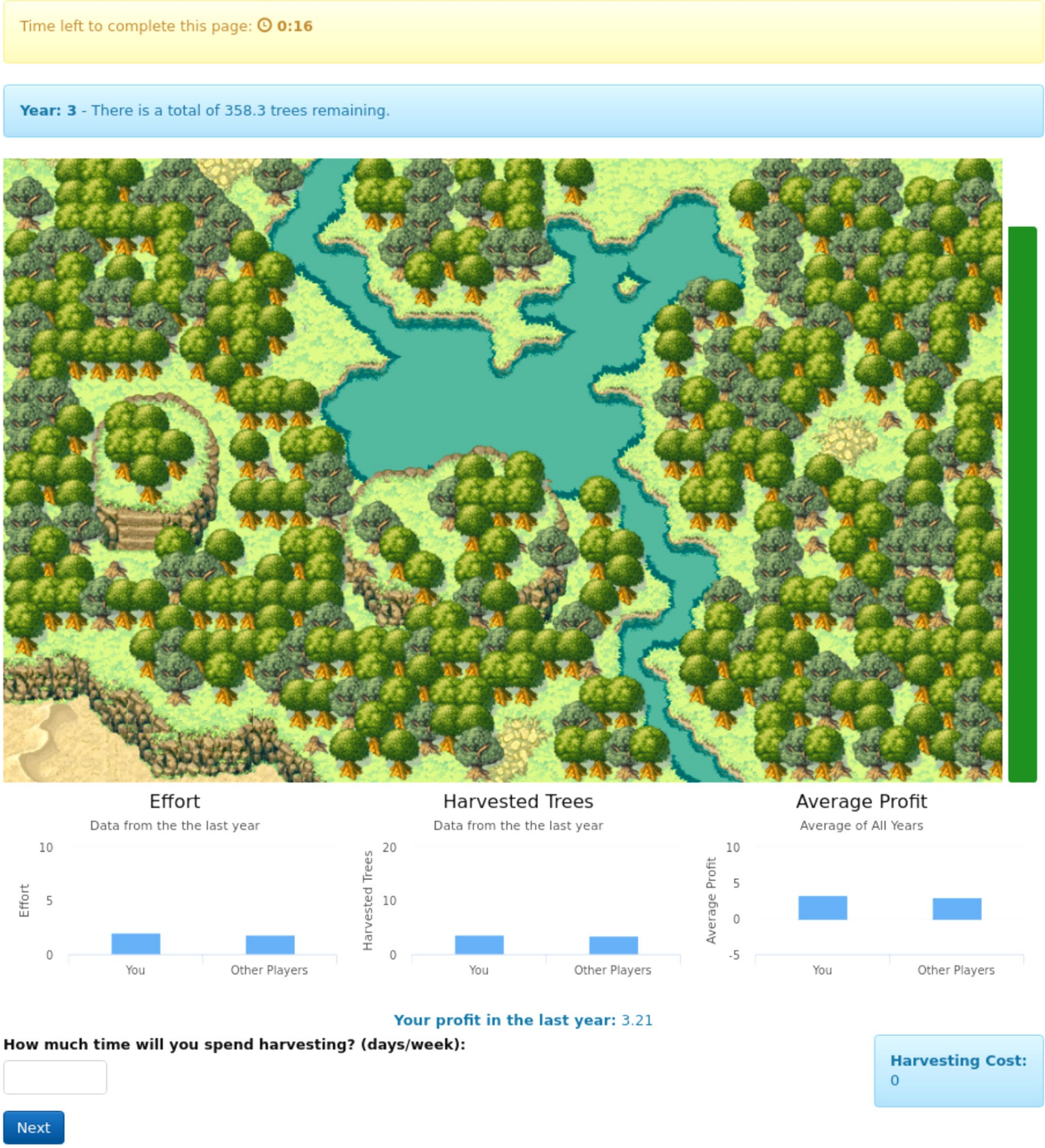

On the screen, you will be able to consult:

• At the top of the screen the year (that is, the current round) and the number of trees that remain in the forest.
• The state of the forest is represented graphically in the drawing, as well as via the bar on the right side.
• The bottom bars respectively show effort over the last year (days of harvest per week), trees harvested over the year, and profit averaged over all the years since the beginning of the game, all referring to you (left) and to the average of your co-players (right).
• Below the bars, shown is the profit you have obtained in the last year. Beware that this number may be negative if the cost surpasses the revenue from harvesting.

With this information, you are again asked to input the amount of time (days/week) you are willing to spend exploiting the resource, between 0 and 7 days/week (decimal numbers allowed). You will have 30 seconds to post your choice.

At the end

When the game finishes, you will be informed of your accumulated profit over all the rounds. This amount will be converted into euros that you will receive in cash.

Please, click ACCEPT to start

Accept

SUPPLEMENTARY NOTE 1

During the game experiment, the computer system passed user inputs to a mathematical model that simulated resource dynamics. Here, we summarize this model's assumptions, list its equations, and graphically illustrate the said dynamics.

The virtual forest's state during the game experiment reflects tree regrowth and logging by participants. With a goal to generate reasonable dynamics, we constructed a simple mathematical model using the following ecologically motivated assumptions. First, regrowth naturally occurs at rate g and is fastest when the current number of trees in the environment, R, is small relative to the carrying capacity, M. If, however, the number of trees gets smaller than a certain critical threshold, R_{c}, regrowth stops due to the Allee effect. Second, the number of logged trees by the ith player, C_i, is determined by this player's effort, T_i, relative to the time needed to find and process a suitable tree, τ. Finding and processing becomes more difficult as the number of trees gets small compared to the carrying capacity. Furthermore, we incorporated basic economic aspects in the form of revenue from selling logged trees at price p, as well as the cost of logging per unit effort, c. Profit π_i is positive if revenue exceeds the cost, and negative otherwise. Model equations arising from these considerations for a total of N players are

$$\frac{dR}{dt} = g\left(M - R\right) H\left(R - R_{\mathrm{c}}\right) - \sum_{i=1}^{N} C_i, \tag{S5a}$$

$$C_i = \frac{R}{\tau M} T_i, \tag{S5b}$$

$$\pi_i = pC_i - cT_i, \tag{S5c}$$

where $H\left(\cdot\right)$ is the Heaviside step function, i.e., zero if the argument is negative and one if it is positive.

Assuming constant effort, the resource state in the long run tends to an equilibrium value (Fig. S2). Denoting this value R', we obtained from Eq. (S5a) that

$$R' = \frac{M}{1 + \frac{1}{gM}\sum_{i=1}^{N}\frac{T_i}{\tau}},$$

which clearly indicates that the number of trees left for cutting in an equilibrium is some fraction of carrying capacity M, where this fraction decreases (resp., increases) with more effort (resp., faster regrowth).

Focusing on proceeds from resource exploitation, we examined conditions that lead to maximum equitable profit. This implies that all players exert the same effort, T^*, and earn the same profit, π^*, such that $\left.\frac{d\pi^*}{dT}\right|_{T=T^*} = 0$. From the last condition, it follows that

$$T^* = \frac{\tau g M}{N}\left(\sqrt{\frac{p}{\tau c}} - 1\right).$$

The corresponding equilibrium resource state is

$$R^* = M\sqrt{\frac{\tau c}{p}}.$$

In the game experiment, as well as in simulations shown in Fig. S2, we used parameter values $g = 0.0504\,\mathrm{d}^{-1}$, $M = 400$, $\tau = \frac{1}{14}$ d, $R_{\mathrm{c}} = 100$, $p = 1$, and $c = 2$. Consequently, $T^* = 2.765$ days per week and $R^* \approx 151$ trees.

SUPPLEMENTARY NOTE 2

We resorted to a clustering analysis to deepen our understanding of the experimental outcomes in terms of player behaviors. In such an analysis, the optimal number of clusters into which the dataset should be partitioned is not a priori known. As many as 11 different optimality measures for addressing this problem are commonly found in literature [9]. Among these, we selected the silhouette method for its conceptual clarity [10]. The silhouette method contrasts cluster cohesion (i.e., how similar data points are to their respective clusters) to cluster separation (i.e., how dissimilar data points are to other clusters). The larger the average silhouette value of the dataset depending on the number of clusters, the better is the given partitioning into clusters. Using the silhouette method on Chinese and Spanish data separately, we first found that the Chinese participants are best partitioned into three clusters (Fig. S3A). The Spanish case is somewhat ambiguous because partitioning into two clusters yields only a marginally larger average silhouette value than partitioning into four clusters (Fig. S3B). A closer inspection of both options reveals that the results are more informative in the context of our game experiment when the Spanish participants are partitioned into four clusters.

We used four quantitative characteristics as a basis for clustering with the idea that these characteristics would reflect behaviors exhibited in each of the two halves of the game experiment. The four characteristics are cumulative efforts and total profits from both the first and the second half of the game taken separately. We surmised that behavioral changes between the two game halves would be of particular interest given that the resource state deteriorates as time passes, causing profits to decline as well. The described arrangement naturally leads to two types of cluster plots per country; these plots are effort vs. profit in the first half (Fig. S4A, B) and effort vs. profit in the second half of the game (Fig. S4C, D).

We found that three prominent player behaviors, aptly termed aggressive, moderate, and timid depending on the exerted effort, shape the fate of resources in both China and Spain. All three behaviors are highly committed to their resource exploitation strategy, and respond very little to the deteriorating resource state (Fig. S4; cf. relative positioning of behaviors and the corresponding efforts in panels A and C, as well as panels B and D). Consequently, profit correlates strongly with effort at the beginning (Fig. S4A, B), but this correlation weakens by the end of the game (Fig. S4C, D). Only when the resource is depleted (i.e., taken beyond the no-recovery threshold) do players substantially change their exploitation strategies, as evidenced by the flipping behavior exclusive to the Spanish participants whose first-half strategy is a mix of aggressive and moderate (Fig. S4B), but the second-half strategy is timid (Fig. S4D).

For a more precise view of how effort changes between the two halves of the game, we also plotted the first-half effort vs. second-half effort cluster plots (Fig. S4E, F). Among the Chinese participants, the aggressive (resp., moderate and timid) behavior is responsible for increasing (resp., constant and decreasing) effort as the time passes (Fig. S4E). Because aggressive players are more abundant than timid ones, the overall effect is as if the Chinese participants compensated for the deteriorating resource by exerting more effort. Among the Spanish participants, by contrast, the aggressive (resp., moderate and timid) behavior is responsible for constant (resp., constant and decreasing) effort as the time passes (Fig. S4F). Coupling this with a drastic effort reduction by flipping players, the overall effect is that the Spanish participants reduce effort in response to the deteriorating resource. Importantly, these observations are in full agreement with the regression results displayed in Fig. 4 of the main text.

SUPPLEMENTARY NOTE 3

With the same set of parameter values applied to all participants from a given country, our model was implicitly designed to capture the collective behavior. Individual differences in the form of propensities to exert and vary effort at random respectively entered the model via participant-specific constant terms called fixed effects and participant-specific residual variances. Fixed effects have considerably larger absolute values among the Chinese participants (Fig. S6A), thus revealing their stronger propensity to exert effort irrespective of the state of the explanatory variables, including the virtual forest's state or efforts posted by others. This result means that non-zero effort is more likely in China than Spain when the number of trees left for cutting is low. Consequently, six Chinese, but none of the Spanish groups kept depleting the resource until the last round of the game (Fig. 2B, C).

A straightforward interpretation of participant-specific residual variances is that they represent individualistic propensities to randomly vary effort. We find that these variances approximately follow the exponential distribution (Fig. S6B, C), thus reflecting a spectrum of individual behaviors. Some participants stick with previous decisions (smaller residual variance), while others tend to explore all possibilities (larger residual variance). Overall, the Chinese participants were more inclined to randomly vary effort, as evidenced by the mean residual variance of 0.4655 as opposed to 0.3617 for the Spanish participants. Aside from this, there is another interesting way to interpret residual variances.

The total data variance comprises contributions from data variances specific to each participant and from mutual covariances. A model without individualized parameters prioritizes collectiveness and is bound to capture the latter contribution. The former contribution is only captured to the extent that individuals mimic the collective, which we quantified by contrasting participant-specific residual variances with the corresponding data variances. We find that residuals carry an average of 20% and 45% less variance than the corresponding data for the Chinese (Fig. S6B) and the Spanish (Fig. S6C) participants, respectively. A greater variance reduction in the latter case indicates that the Spanish participants better mimicked the collective behavior. This result additionally helps to explain the faster resource depletion in Spain than in China (Fig. 2B, C). The stronger the collective, the weaker is the individual resistance to a dominant trend.

SUPPLEMENTARY NOTE 4

The maximum likelihood theory makes fairly strong assumptions that are often violated in practice when the theory is applied to statistical regression modeling. To cope with this problem, the theory has been amended with estimators that are robust to "reasonable" assumption violations. One such example is the sandwich estimator [5] used herein to estimate the covariance matrix and ultimately the 95% confidence intervals for the model parameters. However, when the theoretical assumptions are severely violated, even the robust estimators fail. Gauging the severity with which assumptions are violated is possible via statistical tests [5], but these tests are fairly technical and demand rather elaborate calculations. Bootstrapping is a set of statistical techniques that offer workarounds for these difficulties [11] at a computational cost that is almost negligible with modern computing power.

We resorted to the resampling of residuals in order to probe the probability distributions of the parameter estimators arising in the context of our behavioural regression model. By estimating these distributions, it is possible to detect potential biases in parameter estimates, as well as situations in which relying on asymptotic normality leads to the wrong estimates of confidence intervals. Our datasets, for example, consisted of one time series (50 rounds) for each participant (96 and 90 in China and Spain, respectively), in which case both strong autocorrelations within a time series or strong cross-correlations between any two time series would violate the model assumptions.

Because simple random resampling would erase potential autocorrelations or cross-correlations in the data, we performed the moving block bootstrap [12]. This approach to bootstrapping preserves the potential structure in residuals by dividing them into $T - B + 1$ overlapping blocks of length B, where T is the length of the original time series. The first block then covers residuals 1 to B, the second 2 to $B + 1$, and so on. The resampling is performed by randomly drawing T/B blocks with replacement. To obtain a sufficiently detailed picture of the probed distributions, we performed 1000 bootstrap simulations with $B = 5$ and, due to the lagged predictors, $T/B = 9$ instead of 10.

The described bootstrapping procedure confirms the validity of the behavioral regression model (Fig. S7). We find no evidence of biased parameter estimates. The bootstrap 95% confidence intervals correspond reasonably well to the 95% confidence intervals obtained by means of the sandwich estimator, thus indicating that the model assumptions are not violated for asymptotic normality to become inapplicable.

We additionally tested the statistical significance of the overall model performance. The purpose was to estimate the probability of obtaining a coefficient of determination as large as $R^2 = 0.689$ (in the case of the Spanish data) by pure chance when in fact the null model was true. The null model presumed constant effort supplemented with noise (see the definition of R^2 in the Supporting Methods section above). Even when the null model is true, the behavioral regression model with its multiple degrees of freedom should capture some data variance, but the key question is how much. If the captured data variance as measured by R^2 were on par with the R^2 obtained by fitting the regression model to the original data, then the significance of this regression model would be questionable.

The performance of the behavioral regression model is highly statistically significant (Fig. S8). As intuitively expected given the coefficient of determination as high as $R^2 = 0.689$ for the Spanish data, we find that the probability of obtaining such a large value is minuscule when the null model is true. It is therefore highly improbable that the results of the behavioral regression model (see Fig. 4 of the main text) are a product of pure chance, thus firmly establishing the statistical significance of the model's performance.

In the context of regression diagnostics, we also performed a numerical experiment to test the employed estimator's

robustness to misspecification. Namely, we had no a priori way of knowing what a full set of predictors for our behavioral regression model might look like. It was therefore important to establish that estimating the correct parameter values would still be likely in the absence of a valid or the presence of an invalid predictor. To this end, we created 1000 synthetic datasets using (i) the seven-parameter model as shown in Fig. 4 of the main text in conjunction with (ii) the resampling of residuals as described above. We then attempted to fit to these synthetic datasets an eight-parameter version of the model containing a spurious lag-six predictor. If the estimator employed in our behavioral regression model was truly robust to misspecification, the most likely outcome of such regression attempts would be that the estimated value of the spurious parameter was close to zero, while the estimated values of other parameters were close to their true values.

Numerical simulation strongly suggest that the behavioral regression model is robust to misspecification (Fig. S9). The estimate of the spurious parameter is indeed likely to be close to zero, while the estimates of other parameters are likely to be close to their true values (Fig. S9). There is no evidence of bias. A detail to be aware of is that a non-zero estimate of the spurious parameter seemingly explains some fraction of the data variance, thus reducing the uncertainty in other estimated parameter values. For this reason, it is a good practice to keep in mind the influence of non-significant, non-zero parameters on regression results.

LIST OF SUPPLEMENTARY FIGURES

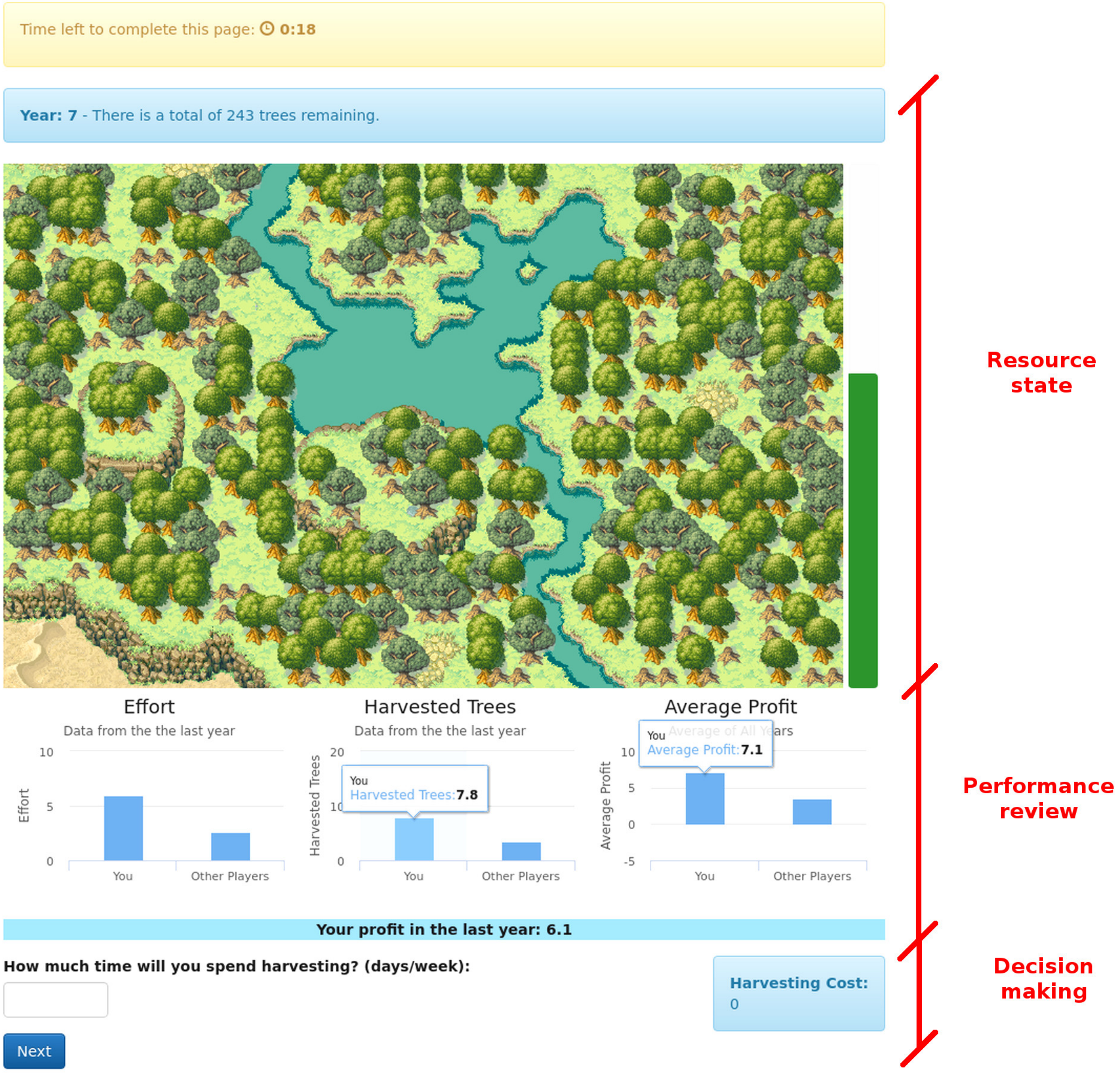

FIG. S1. **Screenshot of the gameplay page**. We divided the gameplay page into three distinct parts: resource state, performance review, and decision making. The resource state part was dominated by a cartoon visualization of the virtual forest, but also contained a status (resp., progress) bar showing the number (resp., fraction) of remaining trees. The performance review part focused on effort, harvest, and profit bar charts with a hover effect such that moving the cursor over any of the bars triggered a tool tip displaying the corresponding numerical value. Lastly, the decision-making part comprised a simple input form asking for the desired effort and a message box that automatically converted effort into the harvesting cost. Final decisions had to be confirmed by clicking the *Next* button. For the actual sessions of the experiment, we used Chinese or Spanish translations of the shown English original.

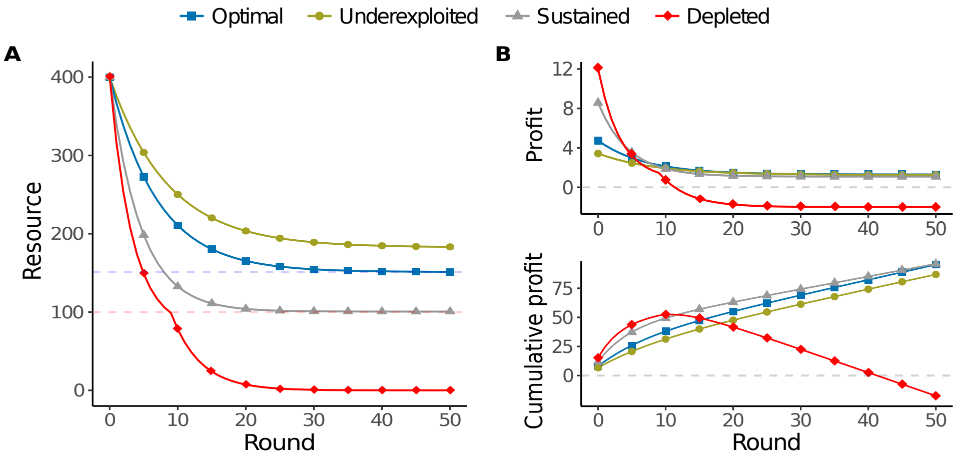

FIG. S2. **Resource state and profits are driven by efforts. A,** Resource dynamics is a combination of regrowth and exploitation. Constant effort leads in the long run to an equilibrium state wherein the number of, in our case, trees left for logging decreases with more effort. If effort is extremely high, regrowth ceases due to the Allee effect and the resource gets depleted. **B,** Profit in a single round is higher when more of the resource is exploited. As the resource approaches an equilibrium, it is optimal in the long run to find the equilibrium that maximizes exploitation, and thus profit. The differences in profit per round among the different equilibria may not be large (upper panel), but they accumulate over time (lower panel). Extremely high effort, by contrast, generates high short-term profits that eventually turn into losses once the resource gets depleted.

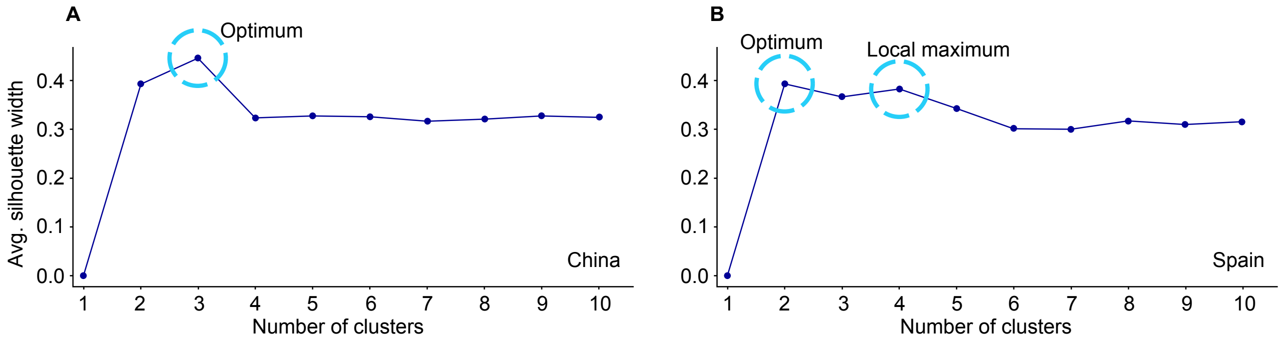

FIG. S3. **Determining the optimal number of clusters with the average silhouette width.** The silhouette value is a measure of how well a data point fits to its own cluster as opposed to other clusters (cohesion vs. separation), ranging from -1 for a poor fit to 1 for a good fit. Averaging silhouette values over an entire dataset produces an aggregate measure, called the average silhouette width, of how well the data have been clustered. This measure is a function of the number of clusters. The best clustering is achieved with the number of clusters for which the average silhouette width is maximal. **A,** For the Chinese data, the optimal number of clusters is three. **B,** For the Spanish data, partitioning into two or four clusters yields nearly an equal average silhouette width. We opted for the latter number because four clusters proved to be very informative in the context of our game experiment (see, e.g., Fig. S4).

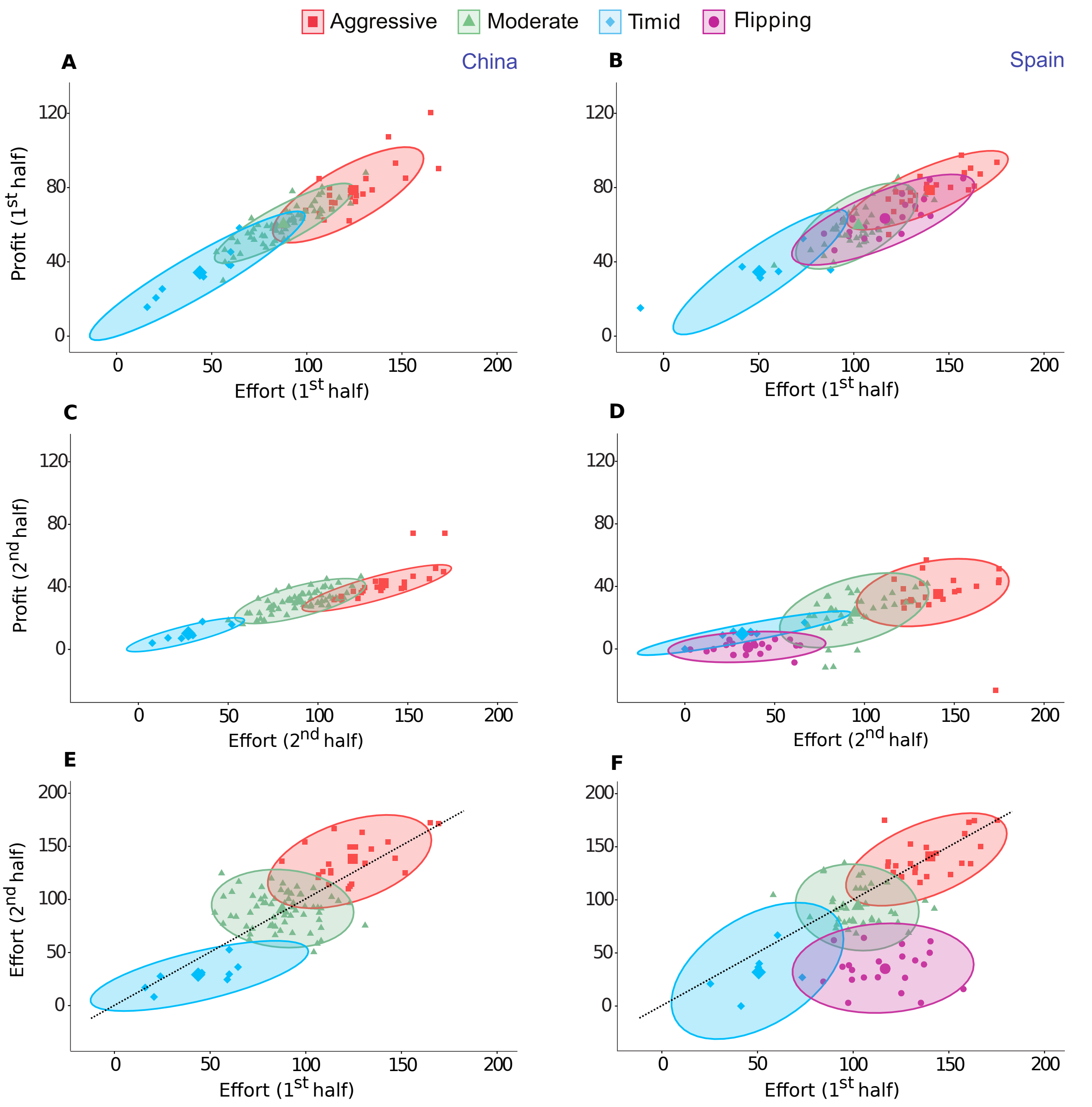

FIG. S4. **Three prominent player behaviors are the same in China and Spain.** Shown are the cluster plots in which axes are the different combinations of clustering characteristics. **A, B,** In the first half of the game, the three prominent behaviors (aggressive, moderate, and timid) act similarly in both countries. The fourth behavior, present only among the Spanish participants (flipping), exhibits the aggressive and moderate characteristics. Profit correlates strongly with effort. **C, D,** In the second half of the game, similarity between the three prominent behaviors still persists. Their mutual relationships (i.e., relative placement in the graphs) is qualitatively the same as in the first half, but the correlation between effort and profit is weaker. Only flipping players change substantially, now resembling the timid ones. **E, F,** Comparing efforts between the two halves of the game, we concluded that the way each of the three prominent behaviors play the game changes very little, and that despite a considerable resource deterioration in the meantime. Only flipping players display a fundamental shift. Generally, the Chinese participants seem to compensate for the declining resource by slightly increasing their efforts (more players situated above the dotted diagonal), whereas the Spanish participants do the opposite. This is fully consistent with the regression results shown in Fig. 4 of the main text.

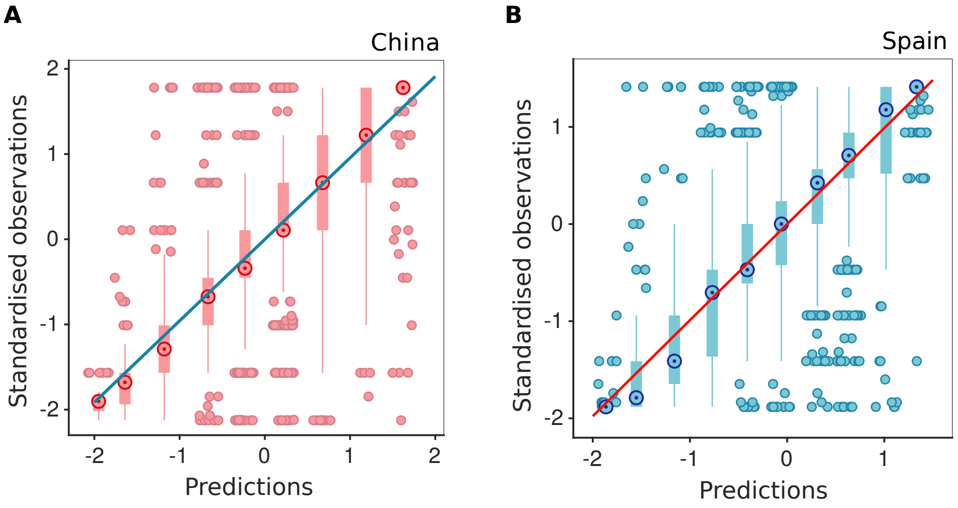

FIG. S5. **Behavioral regression model performs well.** Observation-vs-prediction scatter plots and the accompanying statistics intuitively display and quantify the performance of statistical regression models. In such plots, the scattered points should group around the "diagonal", meaning that the line fitted to these points should be statistically indistinguishable from the line with intercept 0 and slope 1. **A,** For the Chinese data, the intercept is indeed indistinguishable from 0 (estimate -0.0018; 95% CI $[-0.0221, 0.0186]$), but the slope is slightly lower than 1 (estimate 0.9544; 95% CI $[0.9279, 0.9809]$), thus suggesting that the model somewhat overpredicts (resp., underpredicts) low (resp., high) efforts. The coefficients of determination is $R^2 = 0.592$. **B,** For the Spanish data, these minor performance issues disappear because not only the intercept is indistinguishable from 0 (estimate -0.0006; 95% CI $[-0.0193, 0.0180]$), but also the slope is indistinguishable from 1 (estimate 0.9888; 95% CI $[0.9659, 1.0116]$). The coefficients of determination is $R^2 = 0.689$. Due to a large number of data points (>4000 per plot), we grouped them into bins as evenly as possible, and then displayed the medians (circles), the interquartile ranges (boxes), the limits that would encompass 99.3% of normally distributed data (whiskers), and "outliers" (individual points).

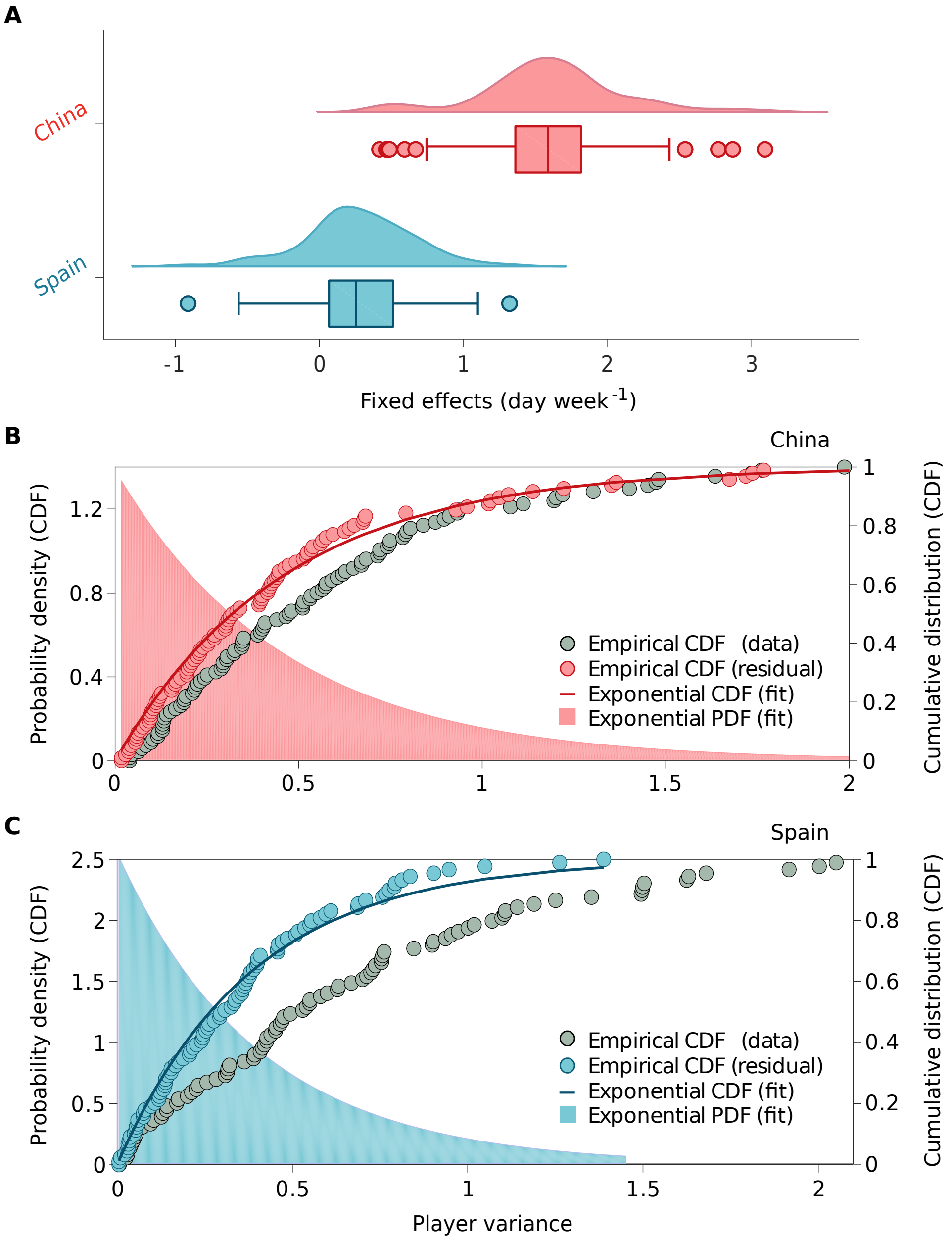

FIG. S6. **Chinese participants manifested stronger individualistic propensities than their Spanish counterparts.** The regression model's implicit focus was on the collective behavior because parameter values remained the same across all participants from a given country. Individual differences entered the model through constant terms called fixed effects and residual variances, both specific to each participant. **A,** Fixed effects have considerably larger absolute values in China than in Spain. This reveals an individualistic propensity to exert effort by the Chinese participants irrespective of the state of the explanatory variables, including the virtual forest's state or efforts posted by others. Here, shown are the kernel-smoothed probability density for fixed effects and the corresponding box plots in which medians, interquartile ranges, limits that would encompass 99.3% of normally distributed data, and outliers are respectively represented by the central vertical line, boxes, whiskers, and circles. **B, C,** In both countries, residual and data variances appear to be drawn from exponential distributions. The means of these distributions for the Chinese participants are 0.4655 (95% CI $[0.3848, 0.5746]$) and 0.5710 (95% CI $[0.4720, 0.7050]$), respectively, indicating a variance reduction of $\approx$20%. The same quantities for the Spanish participants are 0.3617 (95% CI $[0.2970, 0.4505]$) and 0.6635 (95% CI $[0.5447, 0.8262]$), respectively, indicating a reduction of $\approx$45%. A higher residual variance in China reveals an individualistic propensity of the Chinese participants to randomly vary effort irrespective of the state of the explanatory variables.

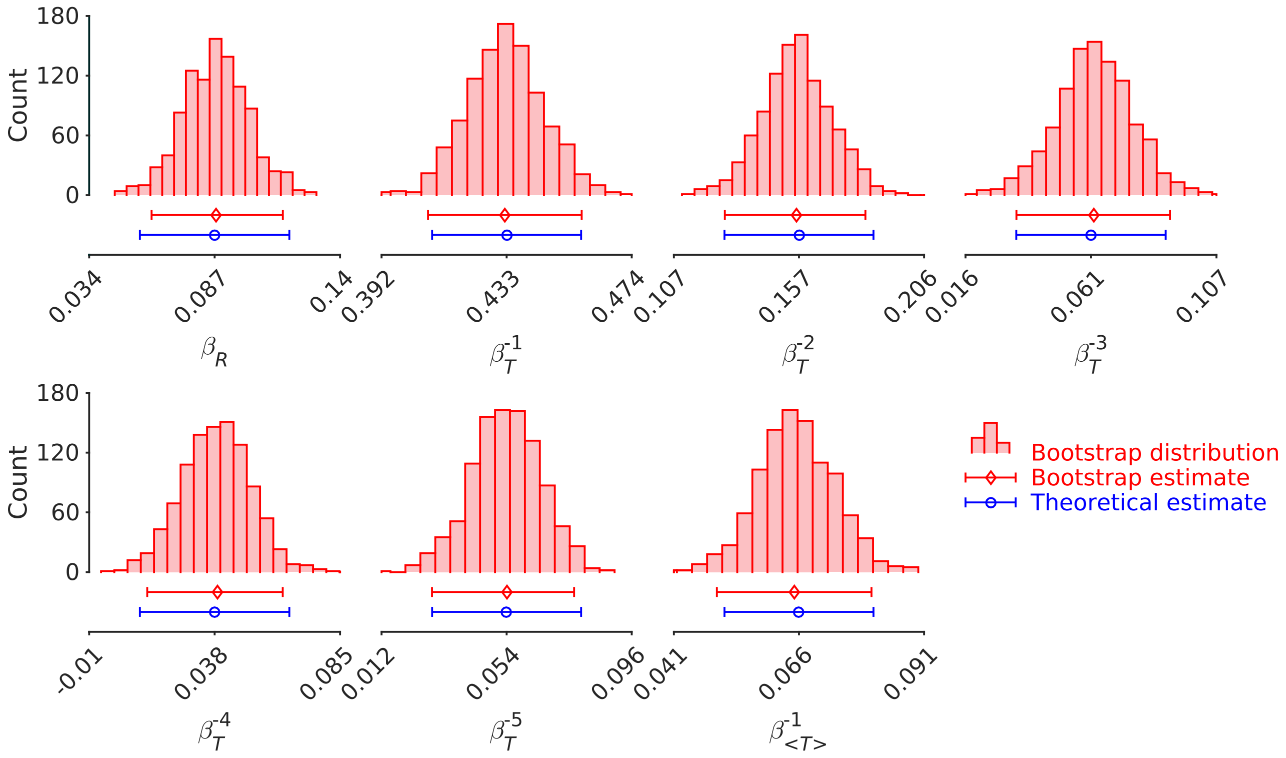

FIG. S7. **Bootstrapping confirms the validity of the behavioral regression model.** Strong assumptions of the maximum likelihood theory are often violated in practice, especially in the context of statistical regression modeling. Although robust estimators circumvent some problems, severe assumption violations may result in biased parameter estimates or overstated parameter significance. An adequate resampling of model residuals, however, allows probing the probability distributions of parameter estimators, thus possibly detecting biases (i.e., a distribution's expectation differs from the true parameter value) or failures of asymptotic normality in constructing confidence intervals (i.e., a distribution's p% interquantile range differs from the p% confidence interval obtained via the covariance matrix). Fortunately, no such problems plague our model as evidenced by a fairly good agreement of bootstrap and theoretical estimates using the Spanish data. Shown are the bootstrap distributions for all parameter estimators (histograms), as well as the corresponding means (red diamonds) and the 95% interquantile ranges (red error bars). Also shown are the maximum likelihood parameter estimates (blue circles) and the corresponding 95% confidence intervals (blue error bars) for an easy comparison.

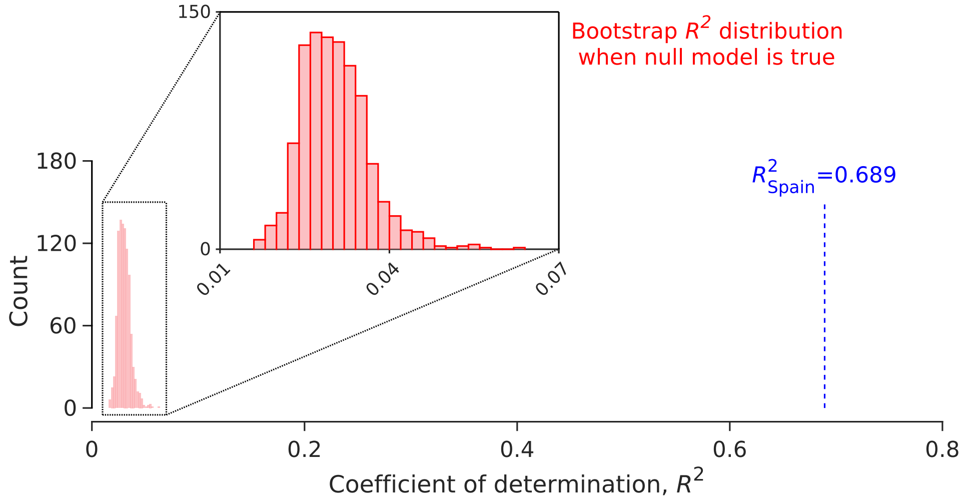

FIG. S8. **Performance of the behavioral regression model is statistically significant.** The histogram represents a bootstrap R^2 distribution for the behavioral regression model when, in fact, the null model (i.e., constant effort with random noise) is true. This distribution shows that the former model with its multiple degrees of freedom explains some data variance even if the data is just noise, yet the performance is poor as intuitively expected. Comparing the 99% quantile of the bootstrap R^2 distribution, $R^2_{99\%}$=0.049, to the value of $R^2 = 0.689$ obtained with the behavioral regression model fitted to the Spanish data, indicates that the model's performance is highly significant. Put alternatively, there would be a negligible probability of obtaining $R^2 = 0.689$ if the null model were indeed true.

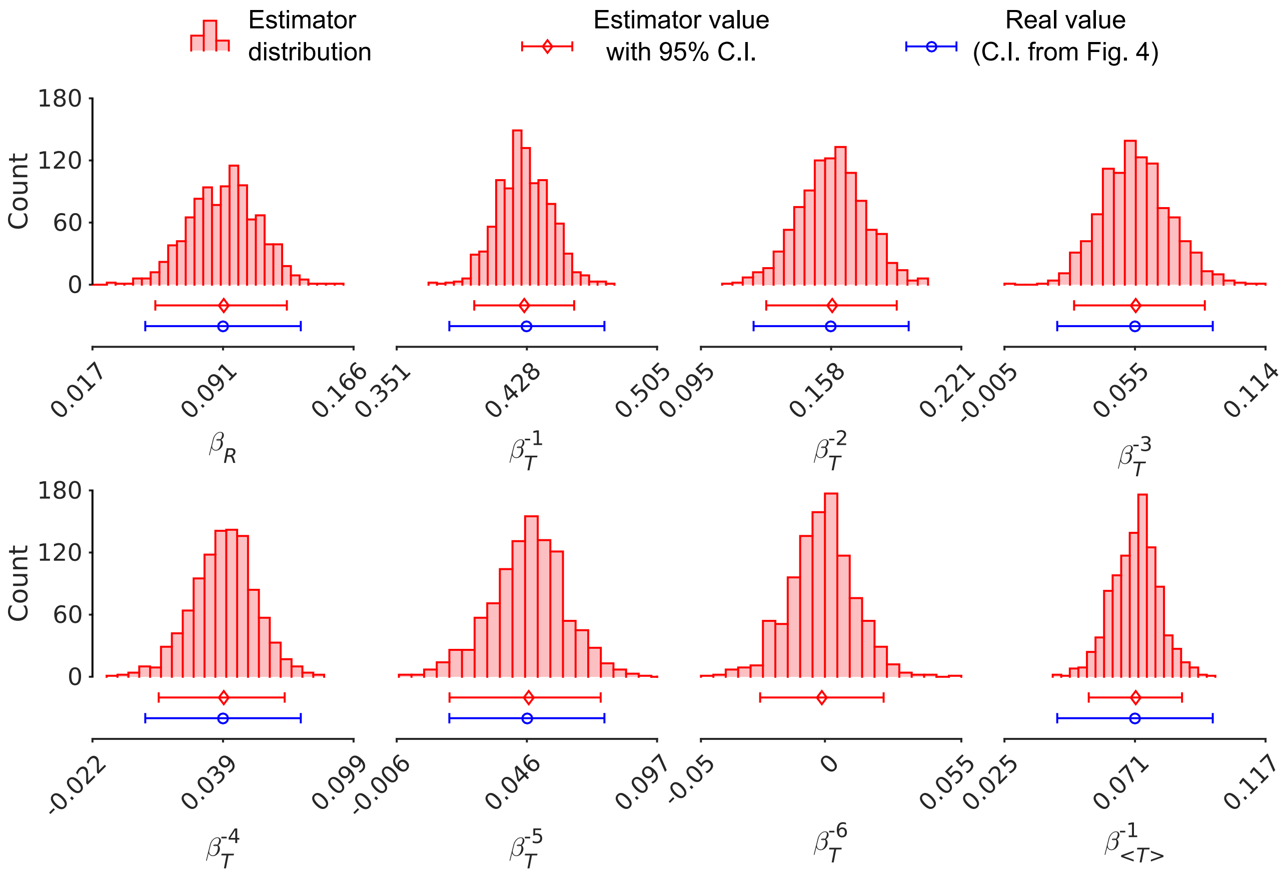

FIG. S9. **Behavioral regression model is robust to misspecification.** Using the same bootstrapping algorithm as in Fig. S7, we generated 1000 synthetic datasets to which we fitted a version of the behavioral regression model with a spurious parameter, β_T^{-6}. The zero value of this parameter is, on average, correctly identified, as are the true values of other parameters. Because the estimator of the spurious parameter is not identically equal to zero, the corresponding predictor seemingly "explains" some of the data variance, thus reducing the uncertainty in other parameters, as evidenced by the upper (red) error bars that are narrower than the lower (blue) ones.

LIST OF SUPPLEMENTARY TABLES

TABLE S1. Basic demographic information on participants.

Country	Participants	Women	Men	Mean age	Minimum age	Maximum age
China	96	37	59	21.5	18	28
Spain	90	48	42	34	18	66

We recruited the Chinese participants from a pool of undergraduate students at the Northwestern Polytechnical University in Xi'an, China. Accordingly, their mean age is relatively young, while their age range is relatively narrow. The Spanish participants better mimic the characteristics of a general population because we recruited them from a broader pool of candidates registered with the Experimental Economics (Nectunt) Lab of the University of Zaragoza. Aside from culture, participants from the two countries differ substantially with respect to confounding factors such as age and education.

TABLE S2. Time-series analysis of the virtual forest's state to determine the presence of significant trends.

	Parameters				Goodness of fit		
Country	c_0	c_1	c_2	c_3	R^2	R^2_{adj}	Resource
China	114.047***	-0.394***	-0.816	0.770***	0.89	0.87	Depleting
China	41.970**	-0.036	-0.365***	0.319	0.70	0.66	Sustained
China	21.193	-0.145	-0.163***	0.376*	0.98	0.98	Sustained
China	30.339**	-0.101*	-0.215***	0.871***	0.94	0.94	Depleting
China	21.944	-0.070	-0.183***	0.085	0.93	0.93	Sustained
China	18.049	-0.075	-0.147***	0.150	0.98	0.98	Sustained
China	83.821***	-0.274**	-0.717	0.305	0.92	0.91	Depleting
China	67.756*	-0.476**	-0.418***	0.280	0.98	0.98	Depleting
China	50.327***	-0.047	-0.430***	0.568**	0.72	0.69	Sustained
China	27.221*	-0.002	-0.229***	-0.216	0.84	0.83	Sustained
China	17.294**	0.047	-0.137***	-0.706***	0.95	0.94	Sustained
China	64.589***	-0.300**	-0.508***	0.516**	0.94	0.94	Depleting
China	80.330***	-0.561***	-0.520***	0.788***	0.94	0.94	Depleting
China	41.448**	-0.049	-0.324***	0.521**	0.77	0.74	Sustained
Spain	30.941*	-0.078	-0.279***	0.398*	0.90	0.89	Sustained
Spain	22.099*	-0.021	-0.190***	0.235	0.82	0.80	Sustained
Spain	26.041**	-0.052	-0.223***	0.515***	0.89	0.87	Sustained
Spain	30.323*	0.036	-0.290***	0.237	0.63	0.59	Sustained
Spain	20.294	0.017	-0.193***	0.327	0.72	0.68	Sustained
Spain	25.381*	-0.063	-0.184***	0.671	0.85	0.83	Sustained
Spain	27.124*	-0.001	-0.227***	0.259	0.74	0.71	Sustained

Denoting with R_t the virtual forest's state at time step t, where $t_0 = 20 \leq t \leq 50$, we fitted model $R_t = c_0 + c_1 (t - t_0) + (1 + c_2) R_{t-1} + c_3 (R_{t-1} - R_{t-2})$ to the time-series data pertaining to the groups who overexploited, but did not deplete the resource. Parameters c_0, c_1, and c_3 are the constant, the trend, and the auto-regressive term, respectively. Parameter c_2 reflects time series stationarity. Star symbols *, **, and *** signify 5%, 1%, and 0.1% statistical significance, respectively. We tested if $c_i \neq 0$, $i = 0, 1, 3$, and $c_2 > -1$. Six Chinese groups kept depleting the resource ($c_1 < 0$) until the end of the experiment. Given more time, these groups would have likely crossed the no-recovery threshold. Interestingly, none of the groups managed to fully reverse the decline and finish with a recovering resource ($c_1 > 0$).

[1] Chen DL, Schonger M, Wickens C. oTree—An open-source platform for laboratory, online, and field experiments. J Behav Exp Finance. 2016;9:88–97.

[2] Hartigan JA, Wong MA. Algorithm AS 136: A k-means clustering algorithm. J Royal Stat Soc C. 1979;28(1):100–108.

[3] Hayashi F. Econometrics. Princeton University Press; 2000.

[4] Amemiya T. Advanced econometrics. Harvard University Press; 1985.

[5] White H. Maximum likelihood estimation of misspecified models. Econometrica. 1982;57(1):1–25.

[6] Nagelkerke NJ. A note on a general definition of the coefficient of determination. Biometrika. 1991;78(3):691–692.

[7] Vuong QH. Likelihood ratio tests for model selection and non-nested hypotheses. Econometrica. 1989;57(2):307–333.

[8] Freedman DA. On the so-called "Huber sandwich estimator" and "robust standard errors". Am Stat. 2006;60(4):299–302.

[9] Liu Y, Li Z, Xiong H, Gao X, Wu J. Understanding of internal clustering validation measures. In: 2010 IEEE 10th International Conference on Data Mining (ICDM). IEEE; 2010. p. 911–916.

[10] Kaufman L, Rousseeuw PJ. Finding Groups in Data. John Wiley & Sons, Inc.; 1990.

[11] Efron B. Better bootstrap confidence intervals. J Am Stat Assoc. 1987;82(397):171–185.

[12] Kunsch HR. The jackknife and the bootstrap for general stationary observations. Ann Stat. 1989;p. 1217–1241.
